# Spatial Genetic Structure and Demographic History of the Wild Boar in the Qinling Mountains, China

**DOI:** 10.3390/ani11020346

**Published:** 2021-01-29

**Authors:** Chaochao Hu, Sijia Yuan, Wan Sun, Wan Chen, Wei Liu, Peng Li, Qing Chang

**Affiliations:** 1 Analytical and Testing Center, Nanjing Normal University, Nanjing 210046, China; huweichen@126.com; 2 Jiangsu Key Laboratory for Biodiversity and Biotechnology, College of Life Sciences, Nanjing Normal University, Nanjing 210046, China; sijiayuanlucas@163.com (S.Y.); sunwan0408@163.com (W.S.); lipeng@njnu.edu.cn (P.L.); 3 College of Environment and Ecology, Jiangsu Open University (The City Vocational College of Jiangsu), Nanjing 210036, China; wanwan0322@163.com; 4 Nanjing Institute of Environmental Sciences, Ministry of Environmental Protection, Nanjing 210042, China; Lw_ecology@163.com

**Keywords:** genetic diversity, genetic structure, phylogeography, qinling mountains, wild boar

## Abstract

**Simple Summary:**

The wild boar is native to the temperate region of Eurasia, which is now one of the most widely distributed mammals worldwide. The recent expansion in the wild boar population has attracted a lot of attention, which may cause great damage to ecosystems. Elucidating the patterns of the population structure, genetic diversity, population origin, and colonization route of wild boar is very helpful in the conservation and management of wild populations. Phylogeographic analysis has proven to be a powerful tool. Here, 82 samples of wild boars in 16 sampling locations were collected in Qinling Mountains (QM). Genetic analysis was conducted based on the mitochondrial control region and nuclear genes. The level of genetic diversity of wild boars in QM was lower than the total population in East Asia, but higher than European population. No obvious phylogeographic pattern were found. The effective population size was under demographic equilibrium in the past.

**Abstract:**

Species dispersal patterns and population genetic structure can be influenced by geographical features. Qinling Mountains (QM) provide an excellent area for phylogeographic study. The phylogeography of Asian-wide wild boars revealed the colonization route. However, the impact of the QM on genetic diversity, genetic structure and population origin is still poorly understood. In this study, genetic analysis of wild boar in the QM was conducted based on the mitochondrial control region (943 bp) and twelve microsatellite loci of 82 individuals in 16 sampling locations. Overall genetic haplotype diversity was 0.86, and the nucleotide diversity was 0.0079. A total of 17 new haplotypes were detected. The level of genetic diversity of wild boars in QM was lower than in East Asia, but higher than in Europe. Phylogenetic analysis showed the weak genetic divergence in QM. Mismatch analysis, neutrality tests, and Bayesian Skyline Plot (BSP) results revealed that the estimates of effective population size were under demographic equilibrium in the past. Spatial analysis of molecular variance indicated no obvious phylogeographic structure.

## 1. Introduction

The Qinling Mountains (QM) are an important geographic and ecological barriers between northern and southern China, which were formed during the Mesozoic and Cenozoic periods extending over 1600 km from east to west in the southern Shaanxi province of China [1]. With limited Late Pleistocene ice-cover, QM provides a good research area for assessing the effect of Pleistocene climatic fluctuations and geologic events on population genetic structure [2]. Because of its diverse habitats and complex topography, the QMs contribute to the biodiversity of the Eastern Asian species by harboring many threatened and endangered animals, such as the golden monkey and giant panda [3,4]. In recent years, many phylogeographical studies discovered that the QM probably served as both a major geographical and ecological barrier for plants and animals [5,6,7]. However, some other results suggested that the QM have no obvious impact on genetic structure [8]. More cases with phylogeographic patterns are needed to make generalizations about the QM impacts on the genetic structure and demographic history of species.

The wild boar (*Sus scrofa*) is a temperate species with diverse groups, and a widespread mammal currently distributed in Europe, North Africa and Asia [9]. In the European wild boar, a latitudinal gradient pattern of intraspecific genetic diversity was observed, despite the fact that human disturbance is common in this species [10,11,12,13]. The current distribution of the mitochondrial genetic pattern of European wild boar was highly influenced by past climatic fluctuations, especially in the Last Glacial Maximum [14,15]. The Southern areas acted as genetic reservoirs in glacial times, and northern areas were mainly recolonized from multi-refugia. The phylogeographical pattern of mtDNA in the European wild boar may result from leading-edge expansion and density-dependent migration processes [11,15].

In East Asia, many previous studies have focused on the origin and the recolonization history of domestic pigs [16,17]. The phylogeography of the Asia-wide wild boar revealed that the wild boar migrated from South-East Asia to South Asia, followed by migration to East and West Asia [18]. Geographical barriers, such as the complex topography of mountains and islands in east and central China, long-term gene flow and population history, play an important role in driving the formation of complex spatial genetic structure of wild boar in East Asia [19]. On a local scale, both Japanese and Korean wild boars are well studied, which has revealed the diverse pattern of genetic diversity and genetic differentiation among wild boar [20,21]. The genetic structure from the QM differed from those from other localities in terms of mitochondrial marker [19]. To date, we have no information on the genetic divergence or population structure of East Asian wild boars in QM, and the population origin, population genetic structure and the degree of admixture have not been well studied.

In this study, we used multiple molecular markers, including the maternally inherited sequences (the complete mtDNA control region, with 943 bp in length), and bi-parentally inherited microsatellite loci (twelve loci), to clarify the spatial genetic structure and historical demography of the wild boar distributed in historically glaciated regions in the QM. Our objectives are as follows: I. to evaluate the genetic status and population genetic diversity in QM; II. to examine the genetic structure of *Sus scrofa* in QM area; III. to discuss the possible origin of wild boar populations in QM by combining our data with the East Asian wild boar mtDNA data from GenBank.

## 2. Materials and Methods

### 2.1. Sampling and DNA Extraction

**Figure 1:**
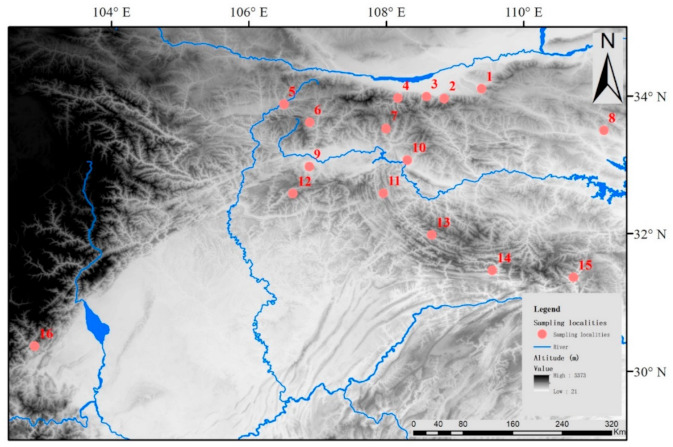
Sampling sites distribution of *Sus scrofa* population in this study. The numbers indicate local populations (see Table 1 for details).

Our experimental procedures complied with the current laws on animal welfare and research in China, and were specifically approved by Nanjing Normal University’s Animal Care and Use Committee (Permit # IACUC-20201206). A total of 82 samples (muscle or ear samples) were collected from 16 localities from 2015 to 2017. According to their geographical distances, topologies and previous phylogeography studies in QM, the sampling localities were grouped into five geographical regions (Table 1, Figure 1).

Samples were stored at −80 °C in our laboratory at School of Life Science, Nanjing Normal University. Total genomic DNA was extracted using commercial DNA isolation kit (QIAGEN, Hilden, Germany) based on the manufacturer’s protocol.

### 2.2. DNA Sequencing and Microsatellite Genotyping

Almost the entire control region (CR) was amplified by polymerase chain reaction (PCR) using two primers [22]. The detailed PCR methods are introduced in Hu et al. (2020). PCR products were then purified and sequenced using the forward primer on an ABI 377 sequencer. Sequences were aligned and checked in MEGA X [23].

Twelve microsatellite loci (Sw632, S0355, S0101, Sw72, IGF, S0005, S0226, S0143, S0225, S0026, S0155, and S0227) were selected, which were previously developed by the International Society of Animal Genetics (ISAG) and Food and Agriculture Organization of the United Nations (FAO) for swine biodiversity studies [24]. PCR products were amplified with fluorescently labeled forward primers (FAM, HEX, TAMRA) and PCR conditions and annealing temperatures followed the sets in the original publications. Amplified products were visualized on an ABI 3730 semiautomated sequencer (PE Applied Biosystems) with internal size marker GeneScan-500 ROX (Applied Biosystems), and the allele data were scored by GeneMarker 2.2.0 (SoftGenetics, LLC., State College, PA, USA).

### 2.3. Genetic Diversity

For mitochondrial DNA data, haplotype diversity (*h*), nucleotide diversity (*π*), and the number of haplotypes were calculated using the program DnaSP 6.12.03 [25].

For microsatellite DNA data, null alleles and large allele dropouts were checked using Micro-Checker 2.2.3 [26]. Molecular diversity indices (mean effective number of alleles, observed heterozygosity, expected heterozygosity, inbreeding coefficients) were calculated using Arlequin 3.5 [27] and FSTAT 2.9.3.2 [28]. Hardy–Weinberg equilibrium (HWE) test and linkage disequilibrium test were performed in Arlequin 3.5.

### 2.4. Phylogenetic Analysis

Two datasets were assembled for phylogenetic analyses: (1) The first dataset used for study of the population genetic structure in QM. The newly sequenced CR (943 bp) were obtained in this study. (2) The second dataset, combined with 680 sequences downloaded from the previous study [19] with 511 bp in length to infer the possible origin of the QM wild boar. The sequences were aligned by using Clustal W implemented in MEGA X [23]. Data format conversion was performed using DnaSP 6.12.03 [25]. The most appropriate model of molecular evolution was selected using MrModeltest 2.3 [29]. For phylogenetic analyses, Bayesian inference (BI) was performed by using the MrBayes 3.2.1 [30]. BI were carried out using the GTR + *G* model of sequence evolution and two independent runs of four Karkov chains (one cold and three heated) over 5 × 10^7^ generations, and every 1000 generations were sampled. The first 25% of sampled trees and estimated parameters were discarded as burn-in. The remaining trees were used to calculate 50% majority rule consensus tree and Bayesian posterior probabilities.

### 2.5. Population Structure and Demographic History

Fu’s *Fs* and Tajima’s *D* neutrality tests were carried out in order to detect evidence of recent population expansion. Fu’s *Fs* [31] and Tajima’s *D* [32] statistics were performed in Arlequin 3.5 [27]. Significance of deviation from neutrality by both *Fs* and *D* indices were assessed by 1000 coalescent simulations.

To infer detailed characteristics of demographic history, we used the Bayesian Skyline Plot (BSP) model by using BEAST 1.8.4 [33]. The molecular clock of 3% substitutions per site per million years was used, which was obtained from the evolutionary rates of mtDNA estimated in previous studies [34,35]. We performed runs with a Markov Chain Monte-Carlo (MCMC) chain length of 1 × 10^8^ generations, and trees were sampled every 1000 generations. This analysis was run in the following parameters: GTR + *G* substitution model without site heterogeneity, relaxed log-normal molecular clock model and tree prior: coalescent Bayesian skyline, with 10 groups and a piecewise-constant skyline model. The first 25% of the generations were discarded as burn-in. All analyses were run to achieve an effective sample size (ESS) at least 200 for all estimated parameters. The effective population size for the posterior distribution of the estimated parameter values was determined using Bayesian skyline plot analysis with the stepwise (constant) model, using TRACER ver. 1.7.1 [36].

STRUCTURE 2.3.2 was used to identify genetically distinct clusters by microsatellite genotypes data, following the admixture model without prior population information [37]. Ten independent runs were carried for different values of *K* from 1 and 10, the MCMC were run for a total of 5 × 10^8^ iterations with discarding the first 5 × 10^7^ iterations as burn-in. Individual assignment probability, Ln P(*D*) and convergence between runs were used to assess the most likely value of K. The most likely number of clusters was estimated according to Evanno et al. [38].

## 3. Results

### 3.1. Genetic Diversity

A total of 78 novel mitochondrial CR sequences were sequenced in this study. After deleting the repeat sequences, we obtained a sequence of 943 base pairs (bp) of the CR, which contained seven singleton variable sites and 21 parsimony informative sites. There are 25 transition sites and three transversion sites, and no indels were observed. The base frequencies were 0.342, 0.263, 0.140 and 0.255 for A, C, G and T, respectively. In total, 17 haplotypes were identified (GenBank accession no. MW417125–MW417141), in which eight haplotypes were only from the single sampling site and nine were shared between sampling sites. Hap3 was the most common haplotype that shared by 24 individuals and exhibiting the widest geographical distribution in nine sampling sites (Table 1). There are three haplotypes (Hap 1, 4, and 7) were shared by five sampling sites, and three (Hap 9, 11, and 8) were shared by two sampling sites.

Diversity indices, haplotype diversity (*h*) and nucleotide diversity (*π*), are shown in Table 2. For the wild boar in QM, *h* was 0.860 (±0.026), and ranged from 0.20 (MQL) to 0.86 (Bashan), and *π* was 0.0079 (±0.0007), and ranged from 0.0006 (MQL) to 0.0076 (Micang), suggesting a moderate haplotype and low nucleotide diversity.

For microsatellite data, 82 individuals were genotyped at 12 loci. Genetic variation at these loci was relatively high. The number of alleles per locus ranged from 8 to 14, with a mean of 10.59. The mean observed heterozygosity was 0.53, and the mean expected heterozygosity was 0.67 (Table 3).

### 3.2. Phylogeography and Population Genetic Structure

**Figure 2:**
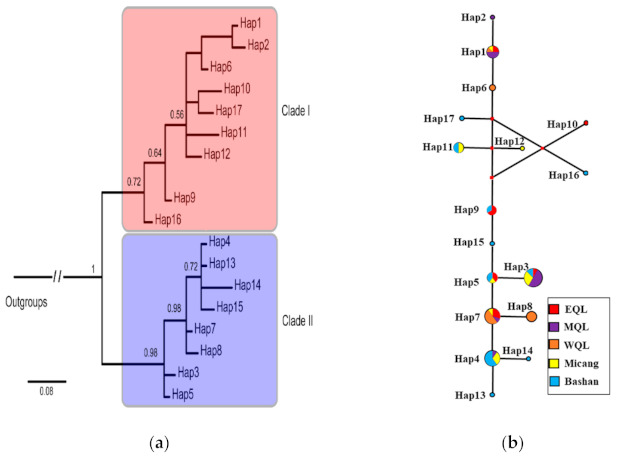
Phylogenetic tree and the median-joining network based on D-loop sequences of wild boar in QM. (**a**) A Bayesian 50% consensus phylogenetic tree for the 17 sampled haplotypes of *Sus scrofa* based on D-loop sequences. Numbers above the tree branches are the posterior probabilities. Numbers indicate haplotype codes (abbreviated to save space: e.g., H1 denotes haplotype 1). (**b**) The median-joining network. Dashes in network represent the corresponding mutation steps. The size of circles denotes the haplotype frequency. Small black circles indicated missing haplotypes that were not observed. The five populations are indicated by different colors. See Table 1 for definitions for EQL, MQL, WQL, Micang and Bashan.

For the first dataset, we obtained 17 haplotypes with 943 bp in length. The phylogenetic tree was estimated with the best-fit model GTR + *G* (−ln*L* = 1950, *p* < 0.001, BIC = 4298) based on the mtDNA CR with two outgroups (*S. barbatus* and *Phacochoerus africanus*). BI analyses produced the topologies with two clades corresponding to Clade I and Clade II, based on mitochondrial haplotypes (Figure 2a). However, the posterior probability of Clade I was relatively lower, with only 0.72. Accordingly, there is no clear genetic divergence in this phylogenetic tree. Haplotypes 6 and 9, located in the basal position of the phylogenetic tree, were from the Clade I, while the basal Haplotypes 3, 5 were distributed in the Clade II (Figure 2a). The median-joining haplotype networks demonstrated a similar phylogeographical pattern to the Bayesian phylogenetic tree (Figure 2b).

**Figure 3:**
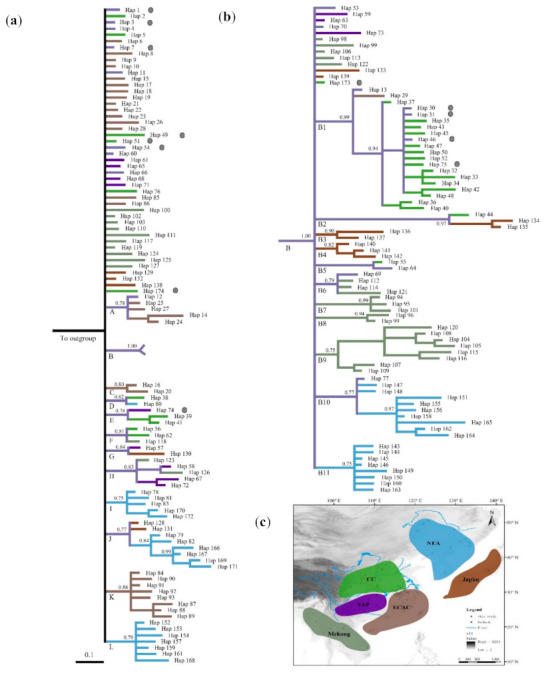
(**a**) Bayesian inferenced tree of East Asian wild boar based on 511 bp mtDNA control region sequences. Numbers above the tree branches are posterior probabilities, and letters under the tree branches represent the name of each clade. The black dots represent the haplotype found in QM area. The color of branches represented samples’ geographical regions corresponding to (**c**). (**b**) The detailed structure of the Clade B. (**c**) Map showing the distribution and sampling localities used in this study. NEA, Northeast Asia; CC, Central China; ECAC, the eastern coastal areas of China; YKP, Yunnan–Kweichow Plateau; Japan, Japan Island; Mekong. The black circles represent samples used in this study; the red circles represent samples downloaded from GenBank.

For the second dataset, we obtained 174 haplotypes with 511 bp in length. The newly sequenced 78 sequences produced 13 haplotypes in this dataset, and only two more haplotypes (Hap 173 and Hap 174) obtained than previous studies (Figure 3) [19]. The sampling localities were grouped into six geographical regions according to their geographical distances and topologies in East Asia (Figure 3c). The results revealed that the phylogenetic tree is defined by a general clade and large polytomy clades (Figure 3). The haplotypes from QM were mainly distributed in the Clade B and general clade, with only one haplotype distributed in Clade E (Figure 3).

**Figure 4:**
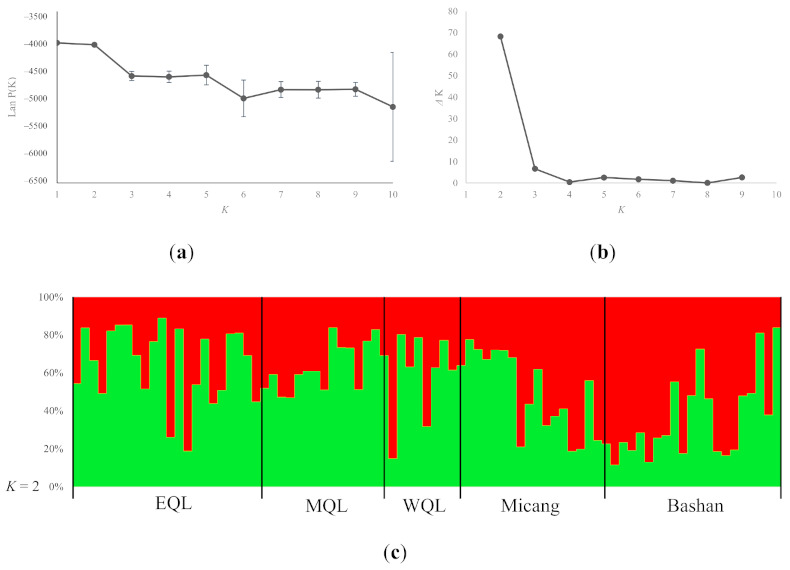
Results of Bayesian model-based clustering in STRUCTURE based on 12 loci. (**a**) The plot of the mean posterior probability LnP(*D*); (**b**) The plot of the values of Δ*K* against *K* values (number of clusters) resulting from 10 runs; (**c**) Bar plots showing Bayesian assignment probabilities for Table 1 for definitions of EQL, MQL, WQL, Micang and Bashan.

For the microsatellite data, we performed the Bayesian assignment analysis to detect population genetic structure. Although no obvious maximum log likelihood of posterior probability was found [LnP (*X*/*K*) = −4085.17] (Figure 4a), the entire dataset yielding the highest Δ*K* statistics output showed a clear maximum at *K* = 2 with Δ*K* = 68 (Figure 4b). When *K* = 2, the diagram showed that some individuals were assigned to two groups with the membership coefficient 40–60% probability (red or green), indicating moderate admixture (Figure 4c). The samples revealed unclear genetic divergence in the microsatellite data.

### 3.3. Historical Demography

We analyzed neutrality tests and goodness of fit to a simulated population expansion and raggedness index of the Clade I, II and the total samples with the mitochondrial DNA data (Table 4). Non-significant negative Tajima’s *D* and Fu’s *Fs* values were obtained for these three sampling pools (Table 4). The significant raggedness index and the mismatch distribution deviate significantly from the expected distribution under a sudden expansion model, which indicated no demographic expansion.

**Figure 5:**
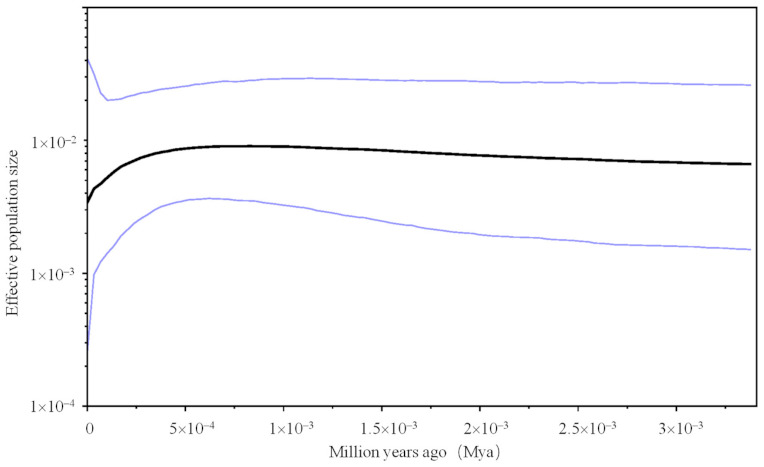
Bayesian skyline plot showing the historical demographic trend of *Sus crofa* in QM for the total samples. Time (*x*-axis) represent in million years and population size (*y*-axis) is measured as the product of effective population size per generation length. Dark lines represent median value inferred NE, blue lines mark the 95% highest probability density (HPD) intervals in all panels.

The BSP based on coalescent theory simulated the fluctuation in populations and showed detailed demographic histories for both clades (Figure 5). ESSs were generally high (>200) for all parameters, indicating good MCMC mixing in the combined chains. For the total samples, the result revealed that the estimates of effective population size were under demographic equilibrium in the past, with a slight increase, which was then followed by an apparent decline in population size dating from approximately 350 years ago.

## 4. Discussion

In this study, a genetic analysis of wild boar in the QM was conducted based on the mitochondrial DNA control region (943 bp) and twelve microsatellite loci of 82 individuals in 16 sampling locations. Overall genetic haplotype diversity is 0.86, and the nucleotide diversity is 0.0079. A total of 17 haplotypes were detected, and the genetic diversity was moderate in East Asia. For mitochondrial data, phylogenetic analysis showed a low genetic divergence of wild boar in QM, and there is no obvious phylogeographic pattern in those areas. Mismatch analysis, neutrality tests, and BSP results revealed that the estimates of effective population size were under demographic equilibrium in the past. For microsatellite data, STRUCTURE identified that most individuals showed admixed genetic structure and revealed no obvious genetic divergence in the microsatellite data.

QM plays on important geographical barriers for the species with low dispersal ability. For amphibians, the study of stream salamander *Batrachuperus tibetanus* revealed three lineages corresponding to the northwestern, eastern and western lineages, which was likely caused by orogenesis of the QM during the late Cenozoic [7]. For reptiles, similarly, the orogeny launched the independent lineage divergence between the northern and southern lineages of endemic Chinese gecko, *Gekko swinhonis* in the QM [5]. For mammals, bayesian analysis of the golden snub-nosed monkey based on 20 microsatellite loci revealed three major clusters which strongly coincide with the major topographical ridge (Main ridge and Huanglongliang ridge) features in the QM, suggesting that gene flow between clusters may be restricted by geographical barriers [3]. However, the study of the golden takin populations based on mitochondrial DNA suggested that no significant geographic genetic diversity across different populations within the QM [8].

In present study, the complete control region of mitochondrial DNA data was used to analysis the genetic diversity and structure of the wild boar in QM. The mtDNA genetic diversity was obvious lower than total of East Asian wild boar, but higher than European wild boar [13,19]. For mitochondrial data, phylogenetics analysis supported that the wild boars in QM may have originated from multiple different genetic clades (Figure 2 and Figure 3), and there is no clear geographical pattern among them (Figure S1). The moderate haplotype diversity and the low nucleotide diversity of the wild boar in QM may be attributed to the relative recent demographic histories. Wild boar populations possibly originated in the island of Southeast Asia, dispersed to East Asia, then radiated into the Indian subcontinent, and finally, spread across East China into North Asia [39]. The colonize route explains the high degree of genetic diversity in the Southeast Asia. The genetic diversities of most sampling pools in East Asia was higher than the European wild boar [19].

Here, we may infer that the wild boar in QM derives from the complicated population history and population expansions in East Asia (Figure 3). In East Asia, environmental changes seemed to be moderate in subsequent climate oscillations [40]. The wild boar has shown successful survival in a wide variety of habitats, with the possible existence of multi-refugia in the inland areas during the Late Pleistocene. Wild boars are widely distributed in East Asia during interglacial period. During glacial episodes, wild boar retreated to refugia. However, the QM are located in an important geographical position with complex topography and variable climates and habitats, and considered to be the center of glaciation in the QM, with limited Late Pleistocene ice-cover [41]. It is likely that wild boar in this area persisted at a relatively stable population size throughout glaciation, and the ecological plasticity may have enhanced its survival [42]. After subsequent warming, the population from refugia dispersed into Qinling areas. The wild boar of East Asian expanded during the interglacial period meet with native populations at QM. The possible gene flow among local populations resulted in the admixture of genetic structure and no obvious phylogeographic structure (Figure 3 and Figure 4; Figure S1). No significant geographic genetic divergence was found across different sampling localities within the QM (Figure 2 and Figure 4). We infer that population admixture and the population history of founder effect played important roles in shaping the spatial genetic structure within wild boars in QM.

We used 12 microsatellite loci to study the genetic diversity and population genetic structure of wild boar in QM. We revealed a moderate level of genetic diversity and a relatively large effective population size, suggesting a relatively high gene flow between populations in QM. In this study, the observed heterozygosity was obviously lower than the expected heterozygosity. Such a lack of heterozygosity may be attributable to the admixture of gene pools from different sources [43]. For microsatellite data, Bayesian assignment analysis revealed the admixed genetic clusters in QM, and the region-specific clusters were not clear. Bayesian assignment analysis and spatial genetic analysis in this study revealed that multiple distinct genetic reservoirs exist in Qinling wild boar, and there may be at least two genetically divergent ancestral sources in QM. The Bayesian spatial genetic structure analysis indicated that the clusters in each sampling sites has changed gradually. Some individuals were assigned to two groups with the membership coefficient 40–60% probability (red or green), indicating moderate admixture (Figure 4). These results indicate the gradual changes in the degree of genetic admixture of the multiple ancestral sources. There is no significant phylogeographic pattern across sampling localities within the QM.

## 5. Conclusions

Our results indicated that the genetic diversity of wild boars in both mitochondrial and microsatellite data of Qinling Mountains was moderate in East Asia, but higher than European wild boar. Phylogenetic analysis showed that there is no obvious phylogeographic structure. The effective population size was under demographic equilibrium in the past. We infer that population admixture and two-times colonization history of founder effect played important roles in shaping the spatial genetic structure of wild boars in QM. This study could be used as a case model for the research into the genetic diversity, genetic origin, and admixture genetic structure of wild animals.

## Figures and Tables

**Table 1:** Sampling information for *Sus scrofa* in this study.

Pop.	Order	Locality (Abbr.)	n	Clade	Haplotype No.	Latitude	Longitude
EQL	1	Lantian, Shaanxi (SAXlt)	1	I	1	34.103	109.388
	2	Weiziping, Shaanxi (SAXwzp)	8	I, II	1, 3, 5, 7	33.963	109.388
	3	Huxian, Shaanxi (SAXhx)	3	I	9–10	33.991	108.845
	8	Xiping, Henan (HNxp)	9	I, II	1–4	33.498	108.586
MQL	4	Chenhe, Shaanxi (SAXch)	1	II	3	33.970	111.170
	7	Foping, Shaanxi (SAXfp)	7	II	3, 7	33.524	108.168
	10	Shiquan, Shaanxi (SAXsq)	2	II	3	33.067	107.998
WQL	5	Fengxian, Shaanxi (SAXfx)	6	I, II	1, 7–8	33.880	108.304
	6	Liuba, Shaanxi (SAXlb)	6	I, II	6–8	33.616	106.510
Micang	9	Nanzheng, Shaanxi (SAXnz)	4	I, II	3, 11–12	32.971	106.884
	11	Zhenba, Shaanxi (SAXzb)	10	I, II	1, 3–5	32.586	106.877
	12	Nanjiang, Sichuan (SCnj)	2	II	3, 7	32.579	107.956
Bashan	13	Chengkou, Chongqing (CQck)	12	I, II	3–5, 11, 14,15	31.979	106.642
	14	Wuxi, Chongqing (CQwx)	3	II	4, 13	31.466	108.659
	15	Xingshan, Hubei (HBxs)	3	I, II	4, 9, 16	31.361	109.544
-	16	danba, Sichuan (SCdb)	1	I	17	30.365	110.723

EQL, East part of Qinling Mountains (QM); MQL, Middle part of QM; WQL, West part of QM; Micang, Micang Mt; Bashan, Bashan Mt; SCdb, Sichuan Basin; n, number of individuals.

**Table 2:** Genetic diversity and neutral test of wild boar population.

Pop.	n	N	*h*(SD)	*π* (SD)	Tajima’D	Fu’s *Fs*
EQL	22	8	0.849 (0.047)	0.0092 (0.0007)	1.869	3.286
MQL	10	2	0.200 (0.154)	0.0006 (0.0005)	–1.562 *	1.225
WQL	12	4	0.742 (0.084)	0.0073 (0.0022)	0.649	4.930
Micang	16	7	0.792 (0.089)	0.0076 (0.0017)	0.338	2.137
Bashan	18	9	0.869 (0.061)	0.0070 (0.0015)	0.326	0.513
SCdb	1	1	/	/	/	/
Total	78	17	0.860 (0.026)	0.0079 (0.0007)	0.936	0.989

EQL, East part of QM; MQL, Middle part of QM; WQL, West part of QM; Micang, Micang Mt; Bashan, Bashan Mt; SCdb, Sichuan Basin; n, number of individuals; N, number of haplotypes; *h*, haplotype diversity; *π*, nucleotide diversity. (* *p* < 0.05).

**Table 3:** Genetic diversity from 6 gene pools by using 12 microsatellite loci.

Pop.	n	*A* ± SD	*H_O_* ± SD	*H_E_* ± SD
EQL	22	11.36 ± 2.01	0.55 ± 0.11	0.67 ± 0.04
MQL	10	8.00 ± 2.32	0.39 ± 0.13	0.52 ± 0.04
WQL	13	10.00 ± 1.79	0.47 ± 0.14	0.66 ± 0.06
Micang	17	10.09 ± 2.17	0.57 ± 0.15	0.69 ± 0.04
Bashan	20	14.00 ± 2.72	0.55 ± 0.15	0.68 ± 0.03
Total	82	10.59 ± 2.16	0.53 ± 0.14	0.67 ± 0.05

n, number of individuals; A, mean effective number of alleles. *H_O_*, observed heterozygosity; *H_E_*, expected heterozygosity; SD, standard deviation.

**Table 4:** Genetic diversity and demographic statistics using mitochondrial data.

	n	N	*h* (SD)	*π* (SD)	Tajima’s *D*	Fu’s *Fs*	SSD	Rag
I	22	9	0.836 (0.060)	0.0050 (0.0004)	0.514	0.040	0.083 ***	0.171 *
II	56	8	0.752 (0.042)	0.0023 (0.0002)	0.276	0.074	0.033	0.077
total	78	17	0.860 (0.026)	0.0079 (0.0007)	0.936	0.989	0.027	0.031

n, number of individuals; N, number of haplotypes; h, haplotype diversity; *π*, nucleotide diversity. SSD, sum of square deviation (goodness-of-fit to a simulated population expansion); Raggedness, raggedness index. (* *p* < 0.05, *** *p* < 0.001).

## Data Availability

Mitochondrial DNA sequence data have been submitted to GenBank (MW417125–MW417141). Microsatellite data presented in this study are available on request from the corresponding author.

## Supplementary Materials

The following are available online at https://www.mdpi.com/2076-2615/11/2/346/s1, Figure S1: Sampling sites and mitochondrial haplotype distribution of *Sus scrofa*. The numbers indicate locality codes (see Table 1 for details). Pie charts represent proportions of each of the two mtDNA clades (I and II) in each sampling site (the phylogenetic clades are given in Figure 2a).
