# Supplementary material for: Spatial Genetic Structure and Demographic History of the Wild Boar in the Qinling Mountains, China
Source: Animals (Basel). 2021 Jan 29;11(2):346. doi: 10.3390/ani11020346 (PMC7912324; doi:10.3390/ani11020346)
Supplement: Supplementary file 1 [file animals-11-00346-s001.pdf]

animals

MDPI

Supplementary Materials: Spatial Genetic Structure and Demographic History of the Wild Boar in the Qinling Mountains, China

Chaochao Hu [1], Sijia Yuan [2], Wan Sun [2], Wan Chen [3], Wei Liu [4], Peng Li [2] and Qing Chang [2,*]

[1] Analytical and Testing Center, Nanjing Normal University, Nanjing 210046, China; huweichen@126.com
[2] Jiangsu Key Laboratory for Biodiversity and Biotechnology, College of Life Sciences, Nanjing Normal University, Nanjing 210046, China; sijiayuanlucas@163.com (S.Y.); sunwan0408@163.com (W.S.); lipeng@njnu.edu.cn (P.L.)
[3] College of Environment and Ecology, Jiangsu Open University (The City Vocational College of Jiangsu), Nanjing 210036, China; wanwan0322@163.com
[4] Nanjing Institute of Environmental Sciences, Ministry of Environmental Protection, Nanjing 210042, China; Lw_ecology@163.com
* Correspondence: Qingchangnj@163.com; Tel.: +86-25-85898176

Citation: Hu, C.; Yuan, S.; Sun, W.; Chen, W.; Liu, W.; Li, P.; Chang, Q. Spatial Genetic Structure and Demographic History of the Wild Boar in the Qinling Mountains, China. *Animals* **2021**, *11*, 346. https://doi.org/10.3390/ani11020346

Academic Editor: Javier Pérez-González and Juan Carranza

Received: 31 December 2020
Accepted: 27 January 2021
Published: 29 January 2021

Publisher's Note: MDPI stays neutral with regard to jurisdictional claims in published maps and institutional affiliations.

Copyright: © 2021 by the authors. Licensee MDPI, Basel, Switzerland. This article is an open access article distributed under the terms and conditions of the Creative Commons Attribution (CC BY) license (http://creativecommons.org/licenses/by/4.0/).

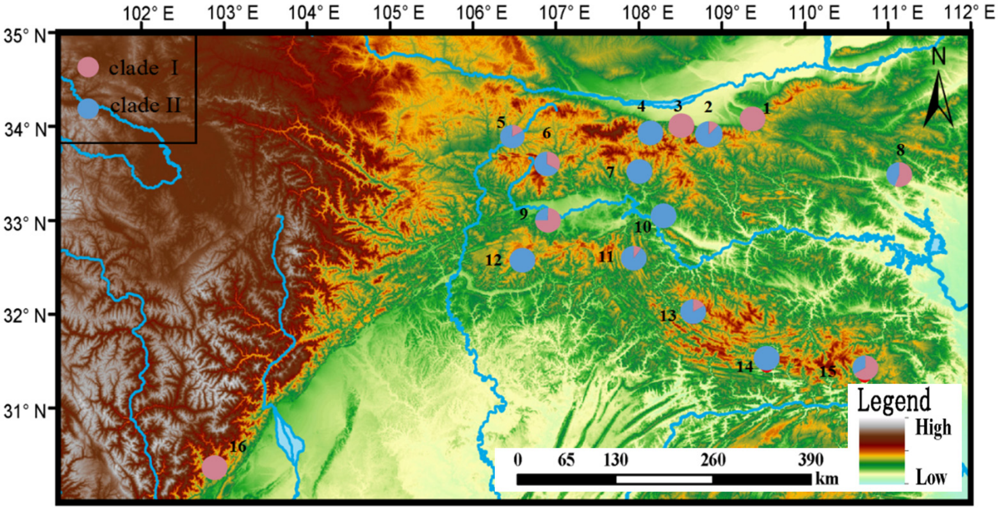

Figure S1. Sampling sites and mitochondrial haplotype distribution of *Sus scrofa*. The numbers indicate locality codes (see Table 1 for details). Pie charts represent proportions of each of the two mtDNA clades (I and II) in each sampling site (the phylogenetic clades are given in Figure 2a).
